# A randomized comparison of health-related quality of life outcomes of dolutegravir versus efavirenz-based antiretroviral treatment initiated in the third trimester of pregnancy

**DOI:** 10.1186/s12981-022-00446-3

**Published:** 2022-06-07

**Authors:** Perez Nicholas Ochanda, Mohammed Lamorde, Kenneth Kintu, Duolao Wang, Tao Chen, Thokozile Malaba, Landon Myer, Catriona Waitt, Helen Reynolds, Saye Khoo

**Affiliations:** 1 Research Department, Infectious Diseases Institute, Makerere University, Hall Lane, P.O Box 22418, Kampala, Uganda; 2 Clinical Sciences, Liverpool School of Tropical Medicine, Liverpool, UK; 3 School of Public Health & Family Medicine, University of Cape Town, Cape Town, South Africa; 4 Molecular and Clinical Pharmacology, University of Liverpool, Liverpool, UK

**Keywords:** HRQoL, Dolutegravir, Pregnancy, Women, HIV, ART

## Abstract

**Introduction:**

Evidence on health-related quality of life (HRQoL) outcomes is limited for new antiretroviral therapies (ART). Dolutegravir-based treatment is being rolled out as the preferred first-line treatment for HIV in many low- and middle-income countries. We compared HRQoL between treatment-naïve pregnant women randomized to dolutegravir- or efavirenz-based ART in a clinical trial in Uganda and South Africa.

**Methods:**

We gathered HRQoL data from 203 pregnant women of mean age 28 years, randomized to either dolutegravir- or efavirenz-based ART. We used the medical outcomes study-HIV health survey at baseline, 24 and 48 weeks between years 2018 and 2019. Physical health summary (PHS) and mental health summary (MHS) scores were the primary study outcomes, while the 11 MOS-HIV subscales were secondary outcomes. We applied mixed model analysis to estimate differences within and between-treatment groups. Multivariate regression analysis was included to identify associations between primary outcomes and selected variables.

**Results:**

At 24 weeks postpartum, HRQoL scores increased from baseline in both treatment arms: PHS (10.40, 95% CI 9.24, 11.55) and MHS (9.23, 95% CI 7.35, 11.10) for dolutegravir-based ART; PHS (10.24, 95% CI 9.10, 11.38) and MHS (7.54, 95% CI 5.66, 9.42) for efavirenz-based ART. Increased scores for all secondary outcomes were significant at p < 0.0001. At 48 weeks, improvements remained significant for primary outcomes within group comparison. Estimated difference in PHS were higher in the dolutegravir-based arm, while increases in MHS were more for women in the efavirenz-based armat 24 and 48 weeks. No significant differences were noted for corresponding PHS scores at these time points compared between groups. Differences between arms were observed in two secondary outcomes: role function (1.11, 95% CI 0.08, 2.13), p = 0.034 and physical function outcomes (2.97, 95% CI 1.20, 4.73), p = 0.001. In the multivariate analysis, internet access was associated with higher PHS scores while owning a bank account, using the internet and longer treatment duration were associated with an increase in MHS scores.

**Conclusion:**

We found no important differences in HRQoL outcomes among HIV-positive women started on dolutegravir relative to efavirenz in late pregnancy. Increases in HRQoL in the first year after delivery provide additional support for the initiation of ART in HIV-positive women presenting late in pregnancy.

*Trial Registration* Clinical Trial Number: NCT03249181

## Introduction

When treatment is initiated in the third trimester of pregnancy, dolutegravir-based antiretroviral therapy (ART) results in greater viral load suppression (less than 50 HIV RNA copies per ml of blood plasma) at delivery compared with pregnant women taking efavirenz-based ART [1]. Dolutegravir-based ART is now a preferred treatment option for HIV infection in mothers, and prevention of perinatal transmission of HIV. However, additional evidence on patient outcomes including health-related quality of life outcomes (HRQoL) is needed for ART regimens in pregnancy.

HRQoL is a patient outcome measure that focuses on addressing multidimensional aspects of health that include patients’ social role, physical status, emotional status, cognitive functioning, and a sense of general satisfaction with life [2, 3]. In addition to commonly used clinical outcomes such as CD4 count and viral load, HRQoL is now an essential outcome in most HIV patient-centered studies [4]. Previous studies found associations between HRQoL outcomes and initiation of antiretroviral therapy among people living with HIV (PLHIV) [5–8]. Some HRQoL evidence exists among HIV patients treated with ART [5–7, 9, 10]. Studies have shown gender differences in HRQoL outcomes among PLHIV for instance, HIV-positive women on ART tend to report poor HRQoL compared with men [11, 12]. However, few studies have examined HRQoL in women and pregnant women living with HIV [13–15]. In addition, pregnancy may lower HRQoL even without HIV infection [16]. Knowledge is scarce on HRQoL in women initiating ART in the third trimester of their pregnancy.

Prolonged use of ART is associated with adverse effects that can potentially impair overall HRQoL [17, 18]. The balance between survival benefits and the adverse effects of using ART on quality of life warrants further research [4, 19]. Adverse events may impair adherence to ART, which in turn could reduce effectiveness for prevention of transmission, and/or prevention of disease progression.

We assessed HRQoL as part of the DolPHIN-2 Study (clinicaltrials.gov registration NCT03249181) that randomized pregnant women initiating ART in the third trimester at the Infectious Diseases Institute and the University of Cape Town in Uganda and South Africa, respectively. We compared the change in HRQoL for women started on dolutegravir- versus efavirenz-based therapy before and after delivery. This knowledge is important to facilitate health policy considerations in programming newer interventions for HIV within sub-Saharan Africa.

## Methods

### Research participants and setting

This study is part of the DolPHIN-2 clinical trial that focused on the treatment efficacy of dolutegravir-based versus efavirenz-based ART initiated in late pregnancy. DolPHIN-2 was a randomized, open-label trial, that recruited pregnant women in South Africa and Uganda aged at least 18 years, living with HIV but are treatment naive and an estimated gestation of at least 28 weeks, initiating ART in the third trimester. Participants were randomly assigned (1:1) to dolutegravir-based or efavirenz-based ART between the years 2018 and 2019. 268 treatment-naïve pregnant women were enrolled in the study. Ethical review committees in South Africa, Uganda, and the UK approved the study. Full details of the clinical outcome study are accessible elsewhere [1].

**Table 1:** Baseline demographic and ART related characteristics

Characteristics	Dolutegravir (n = 82)	Efavirenz (n = 89)	Total (n = 171)
Treatment site			
Uganda	48 (59%)	54 (61%)	102 (60%)
South Africa	34 (41%)	35 (39%)	69 (40%)
Married			
Yes	14 (17%)	9 (10%)	23 (13%)
No	68 (83%)	80 (90%)	148 (87%)
Currently employed			
Yes	35 (43%)	30 (33%)	65 (38%)
No	47 (57%)	59 (66%)	106 (62%)
Phone financial transactions			
Yes	54 (66%)	57 (64%)	111 (65%)
No	28 (34%)	32 (36%)	60 (35%)
Own bank account			
Yes	38 (46%)	41 (46%)	79 (46%)
No	44 (54%)	48 (54%)	92 (54%)
Used internet			
Yes	41 (50%)	44 (49%)	85 (50%)
No	41 (50%)	45 (51%)	86 (50%)
Education level			
Primary	17 (21%)	28 (32%)	45 (26%)
Secondary	50 (61%)	43 (48%)	93 (54%)
Higher/university	2 (2%)	3 (3%)	5 (3%)
Tertiary/vocational	2 (2%)	3 (3%)	5 (3%)
No education	11 (14%)	12 (14%)	23 (14%)
Listen to radio			
At least once a week	68 (83%)	73 (82%)	141 (82%)
Less than once a week	8 (10%)	9 (10%)	17 (10%)
Not at all	6 (7%)	7 (8%)	13 (8%)
Watch television			
At least once a week	64 (79%)	77 (86%)	141 (83%)
Less than once a week	6 (7%)	5 (6%)	11 (7%)
Not at all	11 (14%)	6 (7%)	17 (10%)
Age (years)	27.5 (5.1)	27.5 (5.0)	27.5 (5.0)
Treatment duration (weeks)	56.3 (28.4)	55.9 (30.1)	56.1 (29.2)
Log_10_ viral load (copies per mL)^a^	4.0 (1.1)	4.2 (0.9)	4.1 (1.1)
CD4 at enrollment (cell per µL)^a^	514.9 (283.7)	456.6 (225.2)	484.6 (270)
PHS score	69.9 (11.9)	70.7 (9.2)	70.3 (10.6)
MHS score	81.2 (16.8)	81.5 (15.6)	81.4 (16.1)

Data are n (%) and mean (SD)

^a^At enrollment

### Quality of life measurement

We used the HIV Medical Outcomes Survey (MOS-HIV) to assess HRQoL in trial participants. HRQoL data were collected at three study visits; 4 weeks pre-partum, and at 24 and 48 weeks postpartum. The MOS-HIV is a comprehensive health status measure designed to assess HRQoL in patients living with HIV/AIDS [20, 21]. We used the MOS-HIV 35-item questionnaire that includes eleven dimensions of HRQoL including; general health perceptions (GHP), physical functioning (PH), role functioning (RF), social functioning (SF), cognitive functioning (CF), pain, energy/fatigue, mental health (MH), health distress (HD), quality of life (QoL) and health transition (HT) [22]. We interpreted the survey tool into two local languages of Xhosa and Luganda in South Africa and Uganda respectively. Participant responses to each dimension were aggregated and scores converted to a 0–100-point scale, with 100 indicating the highest achievable health status by a participant [23]. The survey instrument includes two distinct categories of the Physical Health Summary (PHS) and the Mental Health Summary (MHS), based on ten MOS-HIV subscales excluding the health transition dimension [19]. The PHS includes measures of physical functioning, activities, and pain while the MHS includes measures for mental health and psychological functioning [21]. Revicki and others provided evidence from a randomized clinical trial on the reliability and validity of the PHS and MHS scores of the MOS-HIV scales [19, 20, 24].

### Statistical analysis

STATA 13 (StataCorp LP, Texas, USA) and SAS 9.4 (SAS Institute) were used for data analysis. Permuted block randomization (block size of 4), stratified by country was employed and baseline variables were balanced as indicated in Table 1, drawn from the primary clinical outcome study [1]. Power calculations were based on the primary clinical trial endpoint (HIV viral load less than 50 copies per mL at birth) published elsewhere [1]. We used available data with valid HRQoL data from the original trial and no formal sample size calculation was performed in this exploratory analysis of HRQoL outcomes. We employed linear mixed models in this study to estimate differences in HRQoL scores between treatment groups and follow-up visits. During the study, some participants dropped out, resulting in some incomplete observations, and were not imputed but were assumed to be missing completely at random in the mixed model analysis. We specified changes from baseline in HRQoL scores as dependent variables, with baseline measures included as covariates, treatment arm, visit number, and the interaction between visit and treatment as main effects, and subject as a random effect. For example, for the mixed model analysis of change in PHS from baseline, baseline measurements of PHS are treated as a covariate, treatment arm, visit number, and the interaction between visit and treatment as main effects, and subject as a random effect. Further, multivariate analysis was included to determine individual factors associated with HRQoL in late presenting women living with HIV. PHS and MHS were included as dependent variables in the model and baseline measurements were included as covariates (age, employment status, education, viral load CD4 count, and ART treatment duration) and added into the mixed model separately one by one. We based all hypothesis testing on 2-sided tests.

## Results

### Baseline characteristics

Two hundred and three HIV-positive pregnant women with HRQoL information were included in this analysis. 84% (171 out of 203) had baseline socioeconomic and demographic data (Table 1). 71% of the women (145 out of 203) had baseline HRQoL scores. Mean age was 27.5 years (SD ± 5.0) in both treatment groups, with secondary level as the highest education level for 50 (61%) and 43 (48%) of women on dolutegravir and efavirenz, respectively.

### Difference in HRQoL scores at 24 weeks postpartum

Increased scores from baseline in primary outcomes were observed in both dolutegravir and efavirenz groups; PHS (10.40, 95% CI 9.24, 11.55) and MHS (9.23, 95% CI 7.35, 11.10) in the dolutegravir group while PHS (10.24, 95% CI 9.10, 11.38) and MHS (7.54, 95% CI 5.66, 9.42) in the efavirenz group (Table 2). Changes from baseline were significant for all secondary outcomes with p < 0.0001 and p < 0.05 for general health perception and cognitive function. There were no significant differences in PHS and MHS when dolutegravir-based ART was compared to efavirenz-based ART (0.16, 95% CI − 1.47, 1.79) and (1.69, 95% CI − 0.97, 4.35) (Table 3). Overall, no significant differences are observed in primary and secondary HRQoL outcomes when compared between groups, except for role function (1.11, 95% CI 0.08, 2.13) and physical function outcomes (2.97, 95% CI 1.20, 4.73) both with a probability value less than 0.05.

**Table 2:** Summary results from mixed model analysis of primary and secondary outcomes (change from baseline in score) at 24 weeks and 48 weeks by treatment group

Primary and secondary outcomes	Outcomes	Visit (weeks)	Mixed model analysis			
			Dolutegravir		Efavirenz	
			Difference from baseline (95% CI)	p-value^a^	Difference from baseline (95% CI)	p-value^b^
Primary outcomes	Physical health summary	24	10.40 (9.24, 11.55)	< 0.0001	10.24 (9.10, 11.38)	< 0.0001
		48	14.32 (13.08, 15.57)	< 0.0001	13.90 (12.70, 15.11)	< 0.0001
	Mental health summary	24	9.23 (7.35, 11.10)	< 0.0001	7.54 (5.66, 9.42)	< 0.0001
		48	12.50 (10.49, 14.51)	< 0.0001	12.63 (10.63, 14.63)	< 0.0001
Secondary outcomes	Role function	24	6.87 (6.14, 7.60)	< 0.0001	5.76 (5.04, 6.49)	< 0.0001
		48	6.87 (6.48, 7.26)	< 0.0001	6.87 (6.49, 7.26)	< 0.0001
	Physical function	24	16.92 (15.67, 18.17)	< 0.0001	13.95 (12.72, 15.19)	< 0.0001
		48	18.22 (17.51, 18.93)	< 0.0001	18.18 (17.49, 18.88)	< 0.0001
	Social function	24	10.94 (10.15, 11.73)	< 0.0001	11.19 (10.41, 11.97)	< 0.0001
		48	12.53 (12.04, 13.02)	< 0.0001	12.28 (11.80, 12.76)	< 0.0001
	Pain	24	10.91 (8.87, 12.94)	< 0.0001	12.57 (10.54, 14.59)	< 0.0001
		48	15.42 (13.69, 17.14)	< 0.0001	13.71 (12.01, 15.41)	< 0.0001
	General health	24	1.63 (0.55, 2.72)	0.0032	1.88 (0.80, 2.96)	0.0007
		48	4.26 (3.36, 5.17)	< 0.0001	4.27 (3.38, 5.17)	< 0.0001
	Health transition	24	12.07 (8.30, 15.84)	< 0.0001	12.58 (8.81, 16.35)	< 0.0001
		48	11.95 (8.60, 15.30)	< 0.0001	9.79 (6.45, 13.12)	< 0.0001
	Quality of life	24	12.36 (9.28, 15.45)	< 0.0001	9.76 (6.65, 12.86)	< 0.0001
		48	14.89 (12.15, 17.63)	< 0.0001	13.59 (10.85, 16.33)	< 0.0001
	Health distress	24	15.10 (12.45, 17.75)	< 0.0001	11.99 (9.35, 14.63)	< 0.0001
		48	19.33 (17.10, 21.55)	< 0.0001	19.93 (17.74, 22.13)	< 0.0001
	Cognitive function	24	2.30 (0.31, 4.29)	0.0236	4.97 (2.99, 6.95)	< 0.0001
		48	6.60 (4.88, 8.33)	< 0.0001	6.50 (4.79, 8.21)	< 0.0001
	Energy	24	15.49 (13.14, 17.85)	< 0.0001	15.86 (13.51, 18.20)	< 0.0001
		48	22.90 (20.90, 24.89)	< 0.0001	21.43 (19.46, 23.40)	< 0.0001
	Mental health	24	8.57 (6.44, 10.70)	< 0.0001	6.43 (4.32, 8.54)	< 0.0001
		48	11.49 (9.70, 13.29)	< 0.0001	12.26 (10.50, 14.03)	< 0.0001

^a^P-values for estimated differences in dolutegravir

^b^P-vaules for estimated differences in efavirenz

### Difference in HRQoL scores at 48 weeks postpartum

Increases in primary and secondary outcomes from baseline remained significant in both groups (Table 2). The estimated non-significant treatment difference in primary outcomes persisted between groups however; improvements in PHS scores are more for women in dolutegravir relative to efavirenz treatment group. The estimated difference in mean MHS score (− 0.13, 95% CI − 2.97, 2.71) tended to be higher for women using efavirenz relative to dolutegravir (Table 3). Relative to efavirenz treatment group, dolutegravir treated women tended to report more improvement in all secondary HRQoL outcomes except for general health perception and health transition. Overall, differences in primary and secondary outcomes were not statistically different between groups at 48 weeks.

**Table 3:** Summary results from mixed model analysis of primary and secondary outcomes (change from baseline) with repeated measurements at 24 weeks and 48 weeks: between group comparison

Primary and secondary outcomes	Outcomes	Visit (weeks)	n^a^, mean (SD)		Mixed model analysis	
			Dolutegravir	Efavirenz	Difference (95% CI)	p-value^b^
Primary outcomes	Physical health summary	24	86, 11.91 (13.11)	88, 9.02 (10.88)	0.16 (− 1.47, 1.79)	0.8466
		48	74, 15.41 (11.32)	79, 12.62 (9.46)	0.42 (− 1.31, 2.15)	0.6316
	Mental health summary	24	87, 10.56 (15.84)	86, 6.69 (15.26)	1.69 (− 0.97, 4.35)	0.2112
		48	75, 13.41 (16.74)	76, 11.16 (14.34)	− 0.13 (− 2.97, 2.71)	0.9277
Secondary outcomes	Role function	24	89, 9.55 (29.07)	90, 2.78 (21.67)	1.11 (0.08, 2.13)	0.0348
		48	312, 10.90 (30.69)	324, 3.09 (16.40)	− 0.01 (− 0.56, 0.55)	0.9812
	Physical function	24	88, 19.60 (22.53)	90, 11.39 (21.25)	2.97 (1.20, 4.73)	0.0010
		48	308, 20.45 (20.99)	324, 16.05 (16.89)	0.04 (− 0.96, 1.04)	0.9441
	Social function	24	88, 12.73 (25.04)	89, 9.21 (21.33)	− 0.25 (− 1.36, 0.86)	0.6612
		48	304, 14.47 (25.09)	320, 10.50 (20.52)	0.25 (− 0.43, 0.94)	0.4674
	Pain	24	89, 13.34 (28.00)	90, 10.69 (22.37)	− 1.66 (− 4.54, 1.22)	0.2570
		48	312, 17.15 (24.23)	324, 12.19 (19.37)	1.70 (− 0.72, 4.13)	0.1686
	General health	24	89, 1.46 (13.04)	90, 2.67 (12.18)	− 0.25 (− 1.77, 1.28)	0.7521
		48	312, 3.46 (10.83)	324, 5.00 (10.76)	− 0.01 (− 1.28, 1.26)	0.9900
	Health transition	24	88, 9.94 (25.30)	88, 15.06 (27.48)	− 0.51 (− 5.86, 4.83)	0.8512
		48	308, 9.09 (29.90)	316, 12.97 (31.08)	2.16 (− 2.58, 6.90)	0.3707
	Quality of life	24	89, 13.48 (24.73)	88, 9.09 (24.32)	2.61 (− 1.77, 6.99)	0.2428
		48	312, 15.71 (22.00)	316, 12.66 (19.05)	1.30 (− 2.58, 5.18)	0.5096
	Health distress	24	88, 17.61 (30.90)	88, 10.45 (26.19)	3.11 (− 0.64, 6.86)	0.1035
		48	304, 21.51 (30.10)	315, 17.84 (22.72)	− 0.61 (− 3.74, 2.52)	0.7028
	Cognitive function	24	89, 2.58 (18.92)	90, 4.22 (17.17)	− 2.67 (− 5.48, 0.14)	0.0621
		48	312, 6.79 (16.53)	320, 5.94 (17.65)	0.10 (− 2.33, 2.53)	0.9354
	Energy	24	88, 17.27 (21.53)	89, 14.44 (21.60)	− 0.36 (− 3.68, 2.96)	0.8309
		48	307, 24.59 (18.96)	320, 19.81 (20.76)	1.46 (− 1.34, 4.27)	0.3062
	Mental health	24	88, 9.73 (19.17)	90, 5.96 (19.61)	2.14 (− 0.86, 5.14)	0.1622
		48	307, 12.40 (19.68)	324, 11.21 (18.05)	− 0.77 (− 3.29, 1.74)	0.5472

*SD* standard deviation

^a^Number of observations at two visits, each visit compared with baseline

^b^P-value for estimated mean differences between treatment groups at different study visits

In multivariate analysis, several predictors of PHS and MHS scores were identified (Table 4). For PHS, internet use is significantly associated with a 2% increase in PHS score on average (P < 0.05). In the MHS model, owning a bank account, using the internet, watching television at least once a week, and treatment duration are associated with a higher MHS score on average. Compared with uneducated women, women who attained at least primary education are significantly associated with an average decrease in MHS score of 5%. CD4 count is not associated with PHS score while it is significantly associated with lower MHS score in this study.

**Table 4:** Multivariate analysis of factors associated with health related quality of life

Variables	Comparison	PHS		MHS	
		Coefficient (95% CI)	p-value	Coefficient (95% CI)	p-value
Age		− 0.01 (− 0.17, 0.15)	0.8974	0.00 (− 0.26, 0.27)	0.9714
Married	Yes (vs. no)	0.01 (− 2.25, 2.27)	0.9911	− 0.28 (− 3.72, 3.16)	0.8725
Currently employed	Yes (vs. no)	0.42 (− 1.15, 1.99)	0.6002	− 1.55 (− 4.08, 0.99)	0.2291
Phone financial transactions	Yes (vs. no)	− 0.42 (− 2.00, 1.16)	0.5988	1.00 (− 1.59, 3.58)	0.4483
Own bank account	Yes (vs. no)	0.77 (− 0.69, 2.23)	0.2962	5.23 (2.84, 7.62)	< 0.0001
Study site (SA)	SA site (vs. UG site)	0.33 (− 1.15, 1.81)	0.6612	7.53 (5.31, 9.75)	< 0.0001
Used internet	Yes (vs. no)	1.62 (0.19, 3.05)	0.0269	6.65 (4.41, 8.89)	< 0.0001
Listen to radio	At least once a week	0.80 (− 2.00, 3.59)	0.5728	3.24 (− 1.06, 7.55)	0.1384
	Less than once a week	− 0.20 (− 3.62, 3.22)	0.9072	− 4.17 (− 9.48, 1.15)	0.1233
	Not at all	0.00	–	0.00	–
Watch television	At least once a week	0.93 (− 1.37, 3.23)	0.4246	4.68 (1.09, 8.27)	0.0110
	Less than once a week	− 2.19 (− 6.12, 1.73)	0.2706	− 6.12 (− 12.13, − 0.11)	0.0461
	Not at all	0.00	–	0.00	–
Education level	Higher/university	− 2.16 (− 6.63, 2.31)	0.3416	− 1.71 (− 8.90, 5.47)	0.6380
	Primary	− 0.85 (− 3.27, 1.56)	0.4851	− 5.52 (− 9.46, − 1.58)	0.0065
	Secondary	− 1.07 (− 3.23, 1.09)	0.3280	− 1.47 (− 4.94, 1.99)	0.4019
	Tertiary/vocational	1.46 (− 3.03, 5.96)	0.5201	− 0.25 (− 7.82, 7.31)	0.9473
	No education	0.00	–	0.00	–
Viral load		− 0.00 (− 0.00, 0.00)	0.4418	0.00 (− 0.00, 0.00)	0.3030
CD4 count		0.00 (− 0.00, 0.00)	0.3079	− 0.01 (− 0.01, − 0.00)	0.0052
Treatment duration		0.02 (− 0.01, 0.05)	0.1376	0.09 (0.04, 0.13)	0.0003

*PHS* physical health summary, *MHS* mental health summary, *CI* confidence interva, *SA* South Africa, *UG* Uganda

## Discussion

We found significant improvements in HRQoL in both treatment groups from the third trimester of pregnancy to 24 weeks postpartum for all primary and secondary endpoints. These HRQoL outcomes persisted over 48 weeks postpartum in terms of PHS and MHS domains in both treatment groups. We are not aware of any previous analysis of HRQoL for HIV-positive women initiating ART in late pregnancy in a similar LMIC setting. A comparable study in Portugal reported better quality of life in HIV-positive women after childbirth than during pregnancy [25]. Another study in South Africa showed that after 6 months of follow-up in a post-cesarean delivery section, HIV-positive women scored significantly lower on quality of life assessment than HIV-negative women [14]. In the general population of PLHIV, studies have shown a positive impact of ART initiation on HRQoL in Africa [6, 26–29] and outside Africa [21, 30, 31].

We found no significant differences in PHS and MHS scores between-treatment groups at 24 and 48 weeks postpartum. This may be attributable to the comparable clinical effectiveness of dolutegravir and efavirenz. For instance, a previous study in Botswana included 1729 pregnant women initiated on dolutegravir-based therapy and 4593 women initiated on efavirenz-based therapy from government hospitals. Findings showed no statistically compelling difference in the risk for any adverse birth outcome (33.2% vs. 35.0%) and severe birth outcome (10.7% vs. 11.3%) among women using dolutegravir versus efavirenz [32]. Although no significant differences in HRQoL scores were found between treatment groups at 95% CI in the present study, the clinical importance of the small differences is worth considering. A previously published article on the reliability and validity for PHS scores and MHS scores indicated that an over 3-point difference in scores is clinically important [19]. We found no estimated between-group difference equal to or over 3 points for the PHS and MHS at 24 weeks and 48 weeks postpartum.

Between-group differences in secondary endpoints were significant for role function and physical function at 24 weeks postpartum. Participant HRQoL outcomes reflect treatment effectiveness and disease progression [21]. The absence of a significant difference could relate to comparable efficacy between dolutegravir and efavirenz and other non-nucleoside reverse transcriptase inhibitors [33–37].

In the SPRING-1 (phase IIb trial), 205 treatment naïve adult patients were randomized to dolutegravir 10, 25, or 50 mg versus efavirenz 600 mg dose. At 96 weeks, findings revealed that dolutegravir safety profile was more favorable with no dose–response relationship with adverse events; drug tolerance was also generally better with dolutegravir-based dose compared with efavirenz-based dose [38]. Primary analysis from the clinical endpoint study (DoIPHIN-2 RCT) showed non-inferior outcomes for dolutegravir compared to efavirenz on clinical indicators directly linked to participant’s HRQoL. For example, fewer drug-related serious adverse events occurred in the dolutegravir-arm compared to the efavirenz-arm (1.5% vs. 3.8%). More rapid viral load suppression before delivery occurred in the dolutegravir arm compared to efavirenz arm [36].

In the multivariate analysis, we found a negative association between CD4 count and MHS scores. This is surprising since we expect HRQoL to rise with the increase in CD4 count. One African study showed that, although CD4 increased, the quantitative impact on HRQoL changes was minimal in PLHIV [8]. We found no similar study discussing this association in late pregnancy. Two studies in Uganda and South Africa reported no association between CD4 count and HRQoL indicators in HIV general population [39, 40].

Using the internet and watching television was associated with better PHS and MHS scores. Qualitative studies in Uganda and Kenya showed that avoidance and distraction were common copying techniques used by youth living with HIV to prevent poor health outcomes, among these included chatting with friends, watching television, and listening to music [41, 42]. A related study in Europe found a significant association between social support and HRQoL among adult PLHIV [43]. Owning a bank account was associated with higher MHS scores. A study in Uganda found that higher income was associated with a better overall quality of life among PLHIV [26]. Other studies outside Africa showed that employment predicted good overall quality of life for PLHIV [43–45] but this was not identified as a predictor in our study. Most women had lower levels of education and primary level education was negatively associated with MHS in our study. We may attribute this to poor socioeconomic outcomes associated with low education levels, e.g. 62% of respondents reported being unemployed in this study, and employment status influences HRQoL [43, 44].

### Study strength and limitations

Our study used recent data collected as part of the DolPHIN-2 clinical trial study and benefited from robust central data management and statistical team and onsite quality assurance and quality control that ensured accurate collection of data. More than 80% of women entered into this sub-study had at least one HRQoL follow-up assessment. Our study had some limitations. A small sample size could be a limitation when we assess the effects of many covariates on the outcomes. The MOS-HIV is a generic tool for measuring HRQoL in HIV, this may limit its validity in specific HIV diagnostic groups such as pregnant women, and future research should consider using specific tools available. We did not collect information on pregnancy-related covariates and cannot assess whether they are balanced between the treatment arms. As a result, our findings could be subject to possible confounding due to imbalances in some unobserved covariates. Additionally, we gathered and analyzed data in a typical trial environment, we anticipate that some trial-based confounders such as protocol-based HIV care could have biased our findings. However, our study teams received training and study sites designed to minimize any form of bias, we are confident of minimal trial effects on our findings.

## Conclusions

In conclusion, we found no important differences in HRQoL outcomes among women living with HIV, who were started on dolutegravir-based versus efavirenz-based therapy in the last trimester. Increases in HRQoL scores in the first year after delivery provide additional support for the initiation of ART in HIV-positive women presenting late in pregnancy.

## Data Availability

Anonymized de-identified data is available on request to the corresponding author.
